# Ants Can Learn to Forage on One-Way Trails

**DOI:** 10.1371/journal.pone.0005024

**Published:** 2009-04-01

**Authors:** Pedro Leite Ribeiro, André Frazão Helene, Gilberto Xavier, Carlos Navas, Fernando Leite Ribeiro

**Affiliations:** 1 Department of Physiology, University of São Paulo Institute of Biosciences, São Paulo, Brazil; 2 Department of Experimental Psychology, University of São Paulo Institute of Psychology, São Paulo, Brazil; University of Arizona, United States of America

## Abstract

The trails formed by many ant species between nest and food source are two-way roads on which outgoing and returning workers meet and touch each other all along. The way to get back home, after grasping a food load, is to take the same route on which they have arrived from the nest. In many species such trails are chemically marked by pheromones providing orientation cues for the ants to find their way. Other species rely on their vision and use landmarks as cues. We have developed a method to stop foraging ants from shuttling on two-way trails. The only way to forage is to take two separate roads, as they cannot go back on their steps after arriving at the food or at the nest. The condition qualifies as a problem because all their orientation cues – chemical, visual or any other - are disrupted, as all of them cannot but lead the ants back to the route on which they arrived. We have found that workers of the leaf-cutting ant *Atta sexdens rubropilosa* can solve the problem. They could not only find the alternative way, but also used the unidirectional traffic system to forage effectively. We suggest that their ability is an evolutionary consequence of the need to deal with environmental irregularities that cannot be negotiated by means of excessively stereotyped behavior, and that it is but an example of a widespread phenomenon. We also suggest that our method can be adapted to other species, invertebrate and vertebrate, in the study of orientation, memory, perception, learning and communication.

## Introduction

Many ant trails between nest and foraging ground are two-way roads on which outgoing and returning workers meet and touch each other all along [1]. Incoming individuals will recruit their nestmates to take the pheromone-marked path from where they have just arrived [2]–[4]. The orientation ability required of recruited individuals is to follow the scent of the trail, which is kept fresh by the frequent marking behavior of the foragers, as tiny drops of the trail pheromone are laid on the substrate (e.g., ground, tree trunks). As the pheromone is constantly evaporating, the strength of the chemical stimulus in control of the behavior of foragers depends on traffic density. That is how the ants deal with two competing trails of different lengths; the longer route will become less and less appealing as more and more individual workers are attracted by the stronger odor of the shorter path. Yet, once a trail has been well established, they seem unable to take a short-cut [5]–[7]. Such optimization seems impossible, as it simply cannot begin, and even if it began, by chance, with some workers taking the short-cut, it would not compete with the well trodden path, and would be given up [8]. Not all ant trails depend on pheromones. Some species, like the ants of the genus *Cataglyphis*, rely on entirely different orientation systems [9], [10]. The species we have used in this study, however, *Atta sexdens rubropilosa*, is considered as highly dependent on chemical trails. It is known as a leaf cutter and also as a fungus-growing ant, abundant in Brazil. On its trails, hundreds of individuals can be seen traveling back home, with green leaf fragments firmly held in their mandibles, as they pass by hundreds of other foragers on their way to the tree from which their laden nestmates are returning.

Self-organization modeling is a parsimonious approach to complex behavior, currently applied to a very diverse set of functional collective actions, whatever the interacting units. Bird flocks, fish schools, termites building or repairing their nests, human road traffic, neurons working together, seem to be describable by mathematical models based on a few ad hoc hypotheses about individual behavior. Ant trails are considered a remarkable example of a complex outcome brought about by a simple set of interaction rules [11]–[16], and the illustration par excellence of the concept of self-organized systems [8], [17]–[19]. The purpose of our experiments was to test the hypothesis that ants can solve a problem that requires them to switch off their responses to whatever orientation cues they may be using and learn to forage on a unidirectional traffic system. Their failure would mean that, at least in that environment, their responses to orientation cues cannot be inhibited even if they fail to lead them back home or to the food source. Their success would mean the opposite and could also imply the need of some new interaction rules in their self-organization process, like the inclusion of individually learned responses into the models.

For that end, we have developed a method to offer outgoing and returning workers separate one-way routes. Working as a behavioral check valve, the setup renders it impossible for an ant to use the same road in the opposite direction. We have found that workers of the leaf-cutting ant *Atta sexdens rubropilosa* could solve the problem. Not only did they get back home, but they developed a functional unidirectional way of foraging. They could disregard whatever cues they might be using - chemical, path integration, or magnetic - and learned to rely on otherwise secondary visual stimuli in a way that reverses their directional role. Therefore, as an important by-product of our tests, we offer evidence of multimodal navigation in *Atta sexdens rubropilosa*. The key findings however, in our view, are that they could forage on the unidirectional traffic system and that they did so by *reversing* their relationship to visual stimuli, going opposite to the direction where they should be led by them. We suggest that our method can be adapted to other species, invertebrate and vertebrate, in the study of orientation, memory, perception, learning and communication.

## Results

### Experiment 1a. The ants can solve the problem

**Figure 1:**
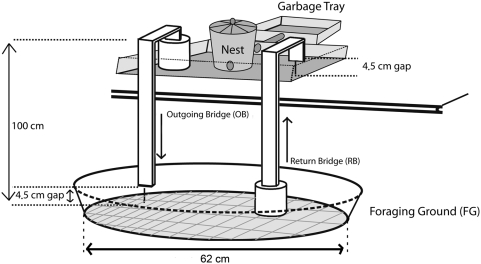
The unidirectional system. In order to forage, a worker must leave the nest, walk toward OB, climb its shorter leg, travel along the crosspiece, go down the longer leg and fall onto FG. After reaching the leaves on FG center, cutting a fragment and grabbing it, it has to take the opposite direction, climb and cross RB, fall from its edge onto the nest area without losing its food load and walk to the nest entrance. Falling is the only way to proceed at the gaps. The bridges are made of wood and its edges are not slippery to ants; they can explore them all along and even extend themselves in the air held by their hind legs without falling.

As described in Fig. 1, the way to stop foraging ants from going back on their steps after getting their food loads - or delivering them at the nest - is to offer them two incomplete bridges connecting nest and food.

In twenty-two of the twenty-five nests in the first experiment, the ants did find the solution: they crossed the outgoing bridge and fell from its end; they cut leaves at the foraging ground, found the return bridge and the leaf fragments to their nests. A foraging routine using the unidirectional path system was developed. After variable test periods - from 20 to 90 days - their fungus gardens were thriving, indicating that the amount of food collected was sufficient to keep them fit. Three nests did not achieve a steady food supply and were discontinued after six days.

### Behavior at the gaps

*Atta sexdens rubropilosa* is not a jumping ant as, for example, *Harpegnathos saltator*, *Myrmecia nigrocincta* and *Gigantiops destructor* [20]. They have never been seen jumping or building living bridges with their own bodies to fill in gaps of any kind between surfaces, as army ants and weaver ants do. Their initial reaction at the gap of both bridges is to stop, explore and go back. They can extend their bodies down in the air with only the two hind legs on the substrate, but they will not jump. They may take brief or long returns, go back to the gap, walk along the edge, reach down again in the air, but they clearly avoid falling. As more individuals reach the spot, lumps of ants are formed, some of them on the bodies of others. Eventually some workers will fall from those lumps. Preliminary tests in which we began with short gaps (1 and 2 cm) on both bridges, instead of beginning with 4.5 cm, showed that some workers could take advantage of the hanging lumps by climbing up on them, thus using the bridges bidirectionally. As we enlarged the gaps, such opportunistic trips ceased altogether and the unidirectional traffic was established. The fall from the edges can be seen as a mechanical consequence of a “keep going” behavior in conflict with an evident “don't let go” command active at any sharp cliff. Working with the ant *Linepithema humile*, a species that is not known to form living bridges, Theraulaz et al. [21] could see hanging lumps of workers, similar to those described above, as they reached the end of an incomplete bridge over an arena with food and water.

### The random route finding hypothesis

**Figure 2:**
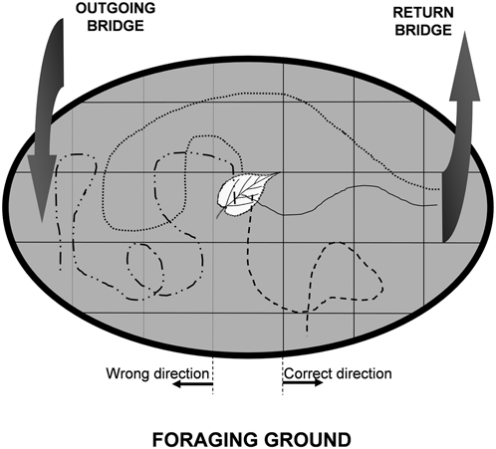
Trip measurements. After cutting and grasping its leaf fragment, the worker begins a return trip; it can be visually followed, and its course, hand-registered on a protocol representing FG, can be measured by a curvometer. Trip outcome will be either successful, when the laden worker climbs up the return bridge, or failed, when it drops its load on FG. The irregular lines on FG floor are actual examples of the four trip types (combinations of direction taken and outcome); trip lengths can go from very short to very long in all four combinations. Such measurements were taken to test the hypothesis that the ants were walking randomly on FG so that their successful trips were not a consequence of oriented behavior.

Effective homing was not a result of random behavior as shown by the return trips of 148 laden workers whose nests had been foraging unidirectionally for at least 20 days (Fig. 2). Right after cutting and grabbing the leaf fragment, most of them (118) left toward the return bridge (RB), instead of going back toward the outgoing bridge (OB); the number of ants taking the direction of the functionally correct location indicates that they did not start their trips randomly (p<0.0001, binomial test) (Table 1). Also, most of the laden workers that started their trips in the correct direction did reach RB (p<0.0001, binomial test), whereas most of those starting in the wrong direction failed to reach RB with their loads (p = 0.0280, binomial test). In the control nests, with two-way bridges, all trips began in the correct direction and most of them reached the two-way bridge (p<0.0001, binomial test) (Table 1). Trip length: the trips of the laden workers that took the correct direction and reached RB were not different from comparable trips of laden workers in the control condition (Mann-Whitney test, p = 0.0515). In fact, they were slightly shorter. The few successful trips begun in the wrong direction were much longer, as evidence of their extended meandering courses, as shown by the comparison between experimental successful laden workers that took the correct direction and those that were successful after taking the wrong direction (Mann-Whitney test, p<0.0001) (Table 1). As failed trips can finish shortly after the laden worker leaves the center of the foraging ground, their median lengths could fall short of adding evidence of the contrast between oriented and disoriented individuals. In practice, however, given the actual numbers, the median length of failed trips begun in the wrong direction did help reveal that contrast when compared with the median length of successful trips begun in the correct direction( Mann-Whitney, p = 0.0211). (Table 1). Direction taken, trip outcome and trip length are evidence that most workers behaved in a correctly oriented fashion while others behaved as if they were lost or wrongly oriented, without implying that any of the four trip types was homogeneously made up of only one of those three behavior patterns (oriented, wrongly oriented or lost) all the time. For instance, an individual laden worker may begin its return trip wrongly oriented (to the outgoing bridge) and then walk sinuously about FG, and finally take an oriented course to the return bridge.

**Table 1:** Direction and length of failed and successful trips in the light and in the dark.

Experimental condition	Measurements	Direction taken and Trip result			
		Correct Direction		Wrong Direction	
		FAILED	SUCCESSFUL	FAILED	SUCCESSFUL
Unidirectional in the light (n = 148)	Proportion of Trips	13.5%	66.2%	13.5%	6.8%
	Median trip length (cm)	54.9 cm	38.1 cm	71.0 cm	100.9 cm
Bidirectional in the light (n = 123)	Proportion of Trips	9.8%	90.2%	0	0
	Median trip length (cm)	25.8 cm	44.2 cm	-	-
Bidirectional in the dark (n = 133)	Proportion of Trips	6.0%	69.9%	9.8%	14.3%
	Median trip length (cm)	90.7 cm	58.2 cm	86.8 cm	147.3 cm

The three measurements – direction taken (correct or wrong), trip length, and trip outcome (failure or success) show that, in the light, most (79.8%) of the experimental (unidirectional) laden workers took the correct direction. Once in the correct direction, their course as far as the return bridge (successful) was as direct and straight as that of the controls (bidirectional in the light). The comparison between the control group in the light (experiment 1) and the control group in the dark (experiment 3) reveals that the absence of light handicapped orientation, but did not stop the ants from foraging effectively.

Although nearly all our nests could solve the problem and forage successfully, the considerable number of ants that took the wrong direction and behaved as if they were lost shows that the condition is a challenge even after 20 days of continuous usage of the system as the only way to get food. It may well be that they could not completely overcome the conflict between their orientation systems and the solution they were capable of finding to the problem. Although 20 days is a considerable time, it may also be that the disoriented workers were newcomers with little or no experience in the system.

### Experiment 1b. A test in the field

The overall result in the field was the same as what we learned in the laboratory. Foragers found the apparatus and solved the problem. The ants steadily took all 20 g of corn offered daily, which meant about 1800 trips per day. Differently from the laboratory nests, the field colony did not depend on our food supply, as it had its usual food sources around.

### Experiment 2. Gradual achievement of effectiveness

Effective foraging does not take place immediately after the nest is submitted to the apparatus. Typically, on the first day, the ants cross the bridges, fall onto the foraging ground and back onto the nest area, but there is very little leaf cutting, and even less leaf transport. On the second day, there is an increase in leaf cutting, but most of the fragments are not taken to the nest; they are dropped anywhere on the floor. From the third day on, the number of successful trips rises steadily, and a routine is reached, with about 400 trips per session, 8 g of leaves being taken to the nest. Qualitatively, we could see that slow development, much longer than in controls, in all our unidirectional nests.

**Figure 3:**
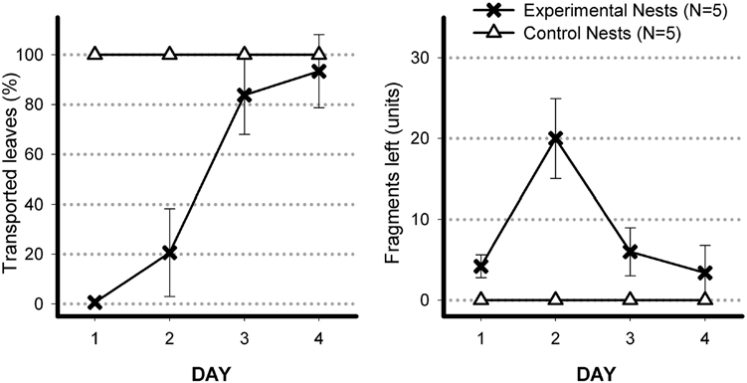
Gradual achievements of effectiveness. Results of the five experimental and the five control nests. Percentages express how much of the total (100%) daily food supply was taken to the nests. The absolute figures express the number of leaf fragments left by the workers on the foraging ground at the end of each session. The two measurements correlate negatively only when all the leaves are cut. If there is no cutting, both of them are zero. They evolve differently, with a peak of left fragments on day 2, showing a three-phase pattern of adaptation to the unidirectional system. The five control groups took all the leaves, leaving not a single fragment, every day.

Quantitatively, we compared five unidirectional with five control nests, by means of a Mann-Whitney test, measuring two variables during days 1 to 4: leaf transport to the nest and leaf fragments left on the foraging ground (Fig. 3). The unidirectional nests cut and transported less than controls on days 1 (p = 0.0039) and 2 (p = 0.0052). That difference decreased on day 3 (p = 0.0539), and disappeared on day 4 (p = 0.3173). They left more fragments than controls on days 1 (p = 0.0186) and 2 (p = 0.0053) but not on days 3 (p = 0.0539) and 4 (p = 0.3173). Examining the course of events, day by day, in the unidirectional nests, it is clear that both leaf transport (Friedman's test, χ2 (3) = 14.76, p = 0.0020) and left fragments (Friedman's test, χ2 (3) = 10.15, p = 0.0174) underwent strong changes. As to leaf transport, days 1 and 2 were different from each other and also from days 3 and 4, which in their turn did not differ from one another (Tukey honest significant difference, days 1×2, p = 0.1759, days 1×3, p = 0.0002, days 1×4, p = 0.0002, day 2×3, p = 0.0003, days 2×4, p = 0.0002, days 3×4, p = 0.7187). Considering left fragments, the second day was different from the other days: the ants cut the leaves but did not take them home (day 1×2, p = 0.0088, day 1×3, p = 0.9679, day 1×4, p = 0.9970, day 2×3, p = 0.0192, day 2×4, p = 0.0063, day 3×4, p = 0.9124).

Therefore, some time consuming process was underway in the unidirectional nests before they reached foraging effectiveness comparable to controls. Working with *Lasius pallitarsis*, Nonacs [22] found that it took foragers no more than minutes to take a different return route when stopped from going back to the outgoing route. The difference between his results and ours may well be due to differences between the two species., as *Lasius pallitarsis* is a more visual ant than *Atta sexdens*. Further investigation with these and other species will help us understand the nature of the problem faced by ants when challenged out of their two-way foraging trails.

### Experiment 3. How they solve the problem: a test in the dark

In nature, this ant forages mostly at night. Differently from other highly visual species, our workers have tiny eyes whose role in orientation has not been hitherto investigated; other *Atta* species have been shown to use their eyes as a supplementary guidance system [23], [24]. In order to investigate the possible role of visual stimuli in their solution to our orientation problem, a darkness-light-darkness experiment was carried out. The five control nests, with two-way bridges, foraged successfully in complete darkness, taking all their leaves everyday, whereas all five experimental nests failed to forage effectively. Their workers went in large numbers to the foraging ground but very little leaf cutting was done. During the three 20 min observation sessions, only six fragments were cut. Successful trips - inferred from leftovers - were very rare, fewer than 20 per day, as compared to the estimated mean of 400 in control nests. On the third day after the light was turned on, the experimental nests were using the system in the effective way described above in the first experiment, taking all the leaves. The light was then turned off and their performance immediately deteriorated back to what it had been in the previous dark session: both leaf cutting and leaf transport stopped altogether. Therefore, although our ants do not need visual cues when foraging as shown by the control nests, the absence of light made it impossible for them to solve the one-way problem in our laboratory conditions. The comparison between control nests in the dark and control nests in the light shows that, although light is not necessary for the development of their bidirectional trails, it seems have an auxiliary role. Control nests in the dark took longer trips than controls in the light, as shown by the comparison of the trip length of successful trips begun in the correct direction in both conditions (Mann-Whitney test, p<0.0001); also, some of the control workers took the wrong direction, while all of the controls in the light took the right direction (Table 1). Such results suggest that the presence of light, though not necessary, improved foraging efficiency.

### Experiment 4. Light source 180° rotation

**Figure 4:**
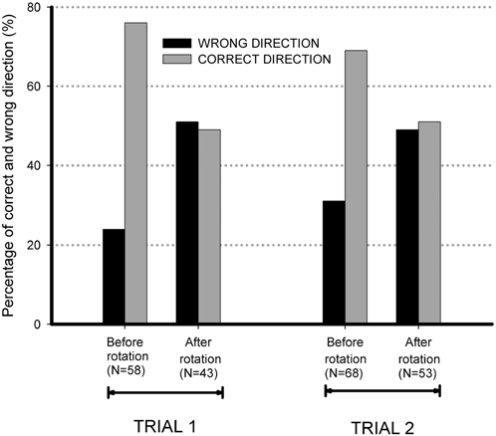
Effect of light source direction on orientation. In both trials, before light source 180 rotation, the ants were foraging effectively in the unidirectional traffic system as shown by the high proportion of trips begun in the correct direction. When the lamp was moved to the opposite side of the apparatus, correct direction fell sharply, as evidence that light direction was being used as a cue to go away from the outgoing bridge toward the return bridge.

In order to help clarify the role of vision in the achievement of unidirectional foraging, we carried out an experiment in which the direction of the light source was controlled. Adding up the two nests and comparing the behavior during the two hours before and after light source rotation (BR and AR, respectively), the results were as follows. In the first trial: total leaf cutting BR, 58; AR, 43 (p = 0.0555). Correct direction as percentage of total leaf cutting, BR 44 (76%) and AR 21 (49%) (testing differences among proportions – Z = 2.59, p = 0.0047 - proportions were compared as an analysis of a contingency table by normal approximation) [25]. In the second trial the results were: total leaf cutting BR, 68; AR, 53 (p = 0.0727). Correct direction as percentage of total leaf cutting, BR 47 (69%) and AR 27 (51%) (testing differences among proportions – Z = 1.85, p = 0.0324, showing a non random distribution among correct and wrong directions). Light rotation significantly disrupted orientation in both trials. The similarity between the two trials shows that during the three-day inter-trial period the ants reoriented themselves to the new light source location. Their foraging effectiveness returned to what it was before the first trial, as shown by the comparison between the proportions of trips in the correct direction before light rotation: 76% in the first and 69% in the second trial (testing differences among proportions - Z = 0.64, p = 0.2601) (Fig. 4).

## Discussion

We hypothesize that in the first, second and third days in the experimental setup, individual ants underwent a change in their orientation responses. Their response to cues leading back to the outgoing bridge was weakened and they learned to respond to light in a reversed way. In their normal bidirectional foraging traffic system, ants will reverse their orientation behavior to any directional cues to which they may be responding, as they begin the journey back the nest. The trails are not chemically polarized [2], [13], but light, the magnetic field, landmarks and occasionally gravity may all inform an ant toward which end of the trail it is heading. They can also gather that information from other ants on the trail, as laden ants will be marching towards the nest. Trails of termites [26], [27] and army ants, *Eciton burcheli* [28] and also Formica [29] have been shown to have lane separation, which seems to be functional in that it allows for on overall higher traffic speed, as it avoids head-on encounters and segregates the slower laden workers from outgoing foragers [30]. Traffic on army ants trails can become unidirectional, when all workers are outbound, and then bidirectional, and later unidirectional again, when all the workers are returning [31]. *Lasius niger* outbound and inbound foraging workers will take turns as they reach a narrow bottleneck on a bridge between nest and food [32]. Such alternations, as well as lane separations, are on the same road. To our knowledge, pheromone-dependent ants have not been seen foraging unidirectionally in the sense of using one route to the food source and a different one back to the nest, the outgoing path never used, not even at its onset, by laden workers, and the return path never used, equally from its onset, by workers going to the food source. Such a pattern would seem impossible given our present knowledge of trail formation, recruitment and orientation, at least among pheromone-dependent central foragers. Our hypothesis holds that vision may have an auxiliary role in the orientation of the foraging workers of our species, and that it is their natural behavior to be responsive to light direction when they go out, and to reverse their orientation to it when they start back to their nests, just as they have to reverse their responses to any other polarized cue they may be using. Therefore, reversing orientation to visual cues would be part of their repertoire. Direction along branching trails can also be provided by negative pheromones that work as no-entry signals[14]. Moreover, trail geometry in itself can give an ant information on which end of the trail lies ahead, as bifurcation angles can help a stranded worker orient itself when it finds the trail [13]. In our setup, the ability to reverse orientation to light was put to use in a surprising way, as they had to learn to move to the opposite side of the foraging ground in order to take the return bridge. In a way, such reversal implies canceling the homing reversal. The fact that light can overcome pheromone attraction in an *Atta* species is in itself quite surprising. Multimodal orientation has been found in several species such as *Formica spp*, [33], [34] and *Camponotus pennsylvanicus*, [35]. The redundancy provided by the usage of more than one modality of cues may serve a number of functions. For instance, one modality can be more precise but the other can allow for higher speed; one can fill in gaps of the other along the trail and they can help an accidentally displaced ant find its way back to the trail. (Harrison et al. 1988), working with *Paraponera clavata*, found that vision and pheromone olfaction, both well developed in that species, can prevail over one another, and that the hierarchical relationship between them depends on previous experience of the ant on the trail. In our experiments, in addition to responding to light direction, the workers may also have adapted their pheromone marking behavior to the new condition. The bridges are functionally unidirectional, but their width offers no constraint to bidirectional traffic, and many workers travel back and forth on them all the time, as they also do on natural trails: they might be marking them as if they were two-way roads. Unloaded *Atta sexdens workers* have been found to reinforce well trodden trails [36]. Such explanation, however, would imply that an individual worker can mark a direction without reaching the destination, so that, when arriving at the edge of the outgoing bridge, it would sense (olfactorily or from the behavior of other workers) that there is food just below, and, stopped by the gap, it would return, mark the way accordingly and recruit other workers, which, in their turn, would be motivated to reach down beyond the gap. Symmetrically, the way home would be marked by ants shuttling on the return bridge, without reaching the nest area. Therefore, on the foraging ground, and on the nest area, the correct direction would gradually overrun the wrong direction in the amount of pheromone in it. Such a pheromone model, however, does not seem to account for the results in the dark, which imply a major role of vision in the solution to the problem, in spite of its clearly secondary role when olfactory orientation is available. The pheromone marking described above is purely conjectural. If it did happen, it is curious that the ants could not go on relying on it when the light was turned off, behaving as though the two modalities could not be integrated. The controls in the dark, however, which were successful but gave clear evidence of being handicapped, suggests that such integration can take place. Further investigation should clear this point.

In Experiment 4, the fact that most trips started in the wrong direction as a consequence of light source rotation supports our hypothesis, and the smaller but considerable number of laden workers that went to the outgoing bridge is consistent with the idea that there is an orientation conflict in the experimental setup.

Our experiments, both with the abrupt procedure of having gaps at the beginning and with the gradual procedure we used in the field by producing the gaps after the ants had begun to get food in the apparatus, reveal a surprisingly flexible behavior. The gaps would seem to entrap the ants in a cul-de-sac, both at the nest area and at the foraging ground. (see Robinson et al. [37] for a discussion on avoidance of being trapped in inferior food sources) We do not suggest that our ants have been facing, in their evolution, the exact problems they solved in these experiments. Their ability to solve them leads us to believe that their plasticity has been shaped in evolution to deal with frequent problems of various kinds in their foraging routine and other collective endeavors. It is our view that, in simple conditions in the natural environment or in simplified laboratory arrangements, their behavior will display to the observer only what is required in order to achieve functional results. The overall schematic description of what they collectively accomplish in such situations may fall short of accounting for the challenges they have to negotiate in actual performances in the natural environment. Substrate irregularities, different materials, weather variations and other constraints may well be responsible for the development of the ability that was put to use in these experiments. We believe that what we have shown is one instance of a widespread property of behavior. In the proper context and within limits, animals behave in ways that, from the point of view of present theory, meet the criteria of problem solving. The study of complex situations in different contexts, and the exploration of the limits of a species in its ability to cope with them, will help develop models with stronger predictive power.

## Materials and Methods

### The nests

They were queenless fragments from five adult *Atta sexdens rubropilosa* laboratory colonies, all of them founded by fertilized females captured on nuptial flight days and taken to the laboratory where they were placed on glass or Plexiglas jars filled with soil. As the colonies grew to maturity, new, empty, 5 l jars were offered, so that, by the time of the experiments, each colony had at least ten jars connected by tubes, all of them with access to the same foraging area, where *Acalypha* leaves were the main food offer. In our experiments, a nest was any one of the jars - except the one where the queen happened to be at the time - taken from the colony, and put on a plastic tray (53 cm×33 cm×8 cm) where the workers had access to a foraging area by simply leaving the jar and walking on the tray. Each nest had several thousand ants. After an adaptation period to the new condition, most nests were strong enough for the experiment.

### Experiment 1

Twenty-five healthy nests, five from each of the five mother colonies, were used. The experiments began by a 24 h food deprivation period, after which the apparatus was put in place with 4.5 cm gaps. For different purposes, in different setups other ant species have been successfully compelled to forage unidirectionally before different authors [22], [38]–[40]. The apparatus used in our experiments (Fig. 1) is made of three wooden pieces painted white. One leg of each bridge is embedded in a supporting plastic box filled with plaster. A daily ad lib supply of *Acalypha* leaves was put on the foraging ground and leftovers were taken out. Observations were discontinued after at least 20 days, over two months in eight cases. Control nests had two-way bridges.

An alternative way of using this method is to take a stepwise approach to gap enlargement similar to what was done in the field test. The apparatus can be put in place without gaps, the ants shuttling across both bridges, and then the two gaps are simultaneously and gradually enlarged until a totally unidirectional traffic is achieved.

### Field test

The field colony was a natural nest in a protected area of the University campus. It had at least 6 “eyes”, openings through which the workers come up to the surface to forage. Three meters from one “eye”, on a place on the ground where there was no trail, we put a plastic box with 20 g of fragmented corn grains. Two continuous bridges - no gaps - longer than but similar in design to those used in our laboratory experiments, were the only way an ant could get into the box as it had an oily wax barrier all along its borders. After the ants found the box and began foraging in it, the end sections of the bridges were pulled up, leaving 2 cm gaps, which were enlarged to 6 cm, 24 hours later. Rodents and birds were denied access to the box by an overall rigid, translucent cover (an upside down large plastic box) that left a thin passage underneath for the ants and also protected the apparatus against rain. A 20 g grain supply was added every morning. Corn fragments were manually counted, so that the number of successful trips could be inferred. The test was discontinued after 10 foraging days.

### Experiment 2

Ten additional naïve nests – five experimental and five controls - had their foraging grounds digitally photographed every 30 min, during the first four nights with the apparatus. Every night, 8 g of *Acalypha* leaves were laid side by side on the center of the foraging ground at 7 pm; 12 hours later, all uncut or partially cut leaves and leaf fragments were taken out. The apparatus remained in place continuously. Using CorelDRAW12, we turned the photos into black and white: leaves and leaf fragments became white and the floor black. Programming MATLAB, we measured how much of the initial white area was missing. The last photo of each session provided the data shown on Table 1.

### Experiment 3

Eight additional naive nests – five experimental and three controls - were taken to a dark chamber and remained there with ad lib *Acalypha* supply for 72 hours, as a pre-experiment adaptation. Any action by the observers was done under red light which was on only during brief human presence. A 24 h deprivation period preceded the placement of the apparatus. Supply procedure was the same as described above for the photographic follow up. After six days in the dark, normal lighting was turned on for three days, and then a new dark period began for four more days. Three 20 min observation sessions of experimental nests were carried out under red light on the fourth day, at a time in the evening when controls were actively foraging. Direction, outcome and length of 133 trips of the control nests were registered.

### Experiment 4

Two naive nests were taken to a dark chamber to ensure that the only light source was a 40 W fluorescent lamp, 2 m away from the center of the foraging ground, sideways to the apparatus. Supply procedure was the same as described above for the photographic follow-up. On the fifth day of unidirectional foraging, two 4-hour observation trials were held, one for each nest, each trial divided into two parts: two hours before and two hours after light source 180° rotation. The lamp was then kept in its new location for three days after which a second 180° rotation was made, preceded and followed by two new 4-hour observation trials. During the three day inter-trial interval, the ants foraged in the apparatus. Both trials began at 9:00 p.m.
